# Adaptive Sampling of the Electrocardiogram Based on Generalized Perceptual Features

**DOI:** 10.3390/s20020373

**Published:** 2020-01-09

**Authors:** Piotr Augustyniak

**Affiliations:** AGH University of Science and Technology, 30-059 Krakow, Poland; august@agh.edu.pl; Tel.: +48-69703-2858

**Keywords:** visual perception, electrocardiogram (ECG) coder, non-uniform sampling, telecardiology, compressed sensing

## Abstract

A non-uniform distribution of diagnostic information in the electrocardiogram (ECG) has been commonly accepted and is the background to several compression, denoising and watermarking methods. Gaze tracking is a widely recognized method for identification of an observer’s preferences and interest areas. The statistics of experts’ scanpaths were found to be a convenient quantitative estimate of medical information density for each particular component (i.e., wave) of the ECG record. In this paper we propose the application of generalized perceptual features to control the adaptive sampling of a digital ECG. Firstly, based on temporal distribution of the information density, local ECG bandwidth is estimated and projected to the actual positions of components in heartbeat representation. Next, the local sampling frequency is calculated pointwise and the ECG is adaptively low-pass filtered in all simultaneous channels. Finally, sample values are interpolated at new time positions forming a non-uniform time series. In evaluation of perceptual sampling, an inverse transform was used for the reconstruction of regularly sampled ECG with a percent root-mean-square difference (PRD) error of 3–5% (for compression ratios 3.0–4.7, respectively). Nevertheless, tests performed with the use of the CSE Database show good reproducibility of ECG diagnostic features, within the IEC 60601-2-25:2015 requirements, thanks to the occurrence of distortions in less relevant parts of the cardiac cycle.

## 1. Introduction

Huge amounts of ECG data are nowadays collected worldwide due to achievements made in the storage of media technology during the last decade. Data compression, as it has been identified in the 1990s, is no longer a necessary condition for the operation of long-term Holter recorders or wireless sensors. Nevertheless, the scientific problem of intelligent adaptive coding remains valid [1,2,3,4,5] and, in the context of cardiac-based home care and surveillance, smart solutions have considerable impact on performance and costs. As such systems commonly use a wireless link, millions of recording hours are difficult to manage and cause high expenses for data transmission [6,7,8]. 

The ECG is sampled with a constant frequency, mainly for the reason of commodity, despite the full bandwidth of the data stream being used only for a very limited period within the QRS complex (of a typical duration of 100 ms). In the remaining part of the heartbeat, the discrete time series is significantly oversampled causing high correlation of neighboring samples unless blurred with noise. A family of short-time decorrelation techniques uses this feature for signal compression (e.g., [9,10]). The oversampled sections are used as a reference for local measurement of noise level (e.g., [11]) or as a host of watermark data (e.g., [12]). A class of algorithms perform compressed sensing based on local statistics (e.g., [13,14,15]) or adaptive sampling based on the mutual data dependence (e.g., [16,17]) but with no regard for distribution of medical relevance in the record. 

In previous research projects we have studied the irregular distribution of medical information in the ECG record with use of various methods: local bandwidth of ECG waves [18], susceptibility of diagnostic result to signal distortion caused by a local data loss, and local conspicuity of the ECG trace [19,20]. Results reported in the latter work include a generalized quantitative estimate of local temporal distribution of the electrocardiogram medical relevance, here referred to as generalized medical relevance function (gMRF). This function is briefly recalled hereafter and proposed as a background for the adaptive ECG sampling technique presented in this paper. 

Due to non-uniform distribution of diagnostic information in the ECG, simple metrics for time series comparisons such as signal to noise ratio (SNR) or percentage root-mean-square difference (PRD) do not adequately represent the degradation of quality of diagnostic results. For the same reasons the lossy compression of the ECG is distrusted [3,8,10], and currently not allowed in clinical applications. At the same time, various techniques for ECG recording with constant sampling frequency ranging from 125 to 1000 Hz are used depending on the medical goals and provide either more concise or more detailed background for diagnostic analyses. This justifies the seamless adaptation of the sampling frequency in relation to the patient status and—like in case of the proposed method—with progression of the cardiac cycle. 

Different phases of the cardiac cycle can be distinguished in the surface-recorded ECG as a representation of sequence of cell action potentials in the heart conduction system and myocardium. Due to different electric properties of the conducting tissue, waves representing the progression of the cardiac cycle show different bandwidths as the stimulus travels through the heart. They also show significant variability in duration on a beat-to-beat basis not directly related to the RR interval even in normal rhythms. In case of abnormal rhythms, the order of waves in the sequence may also be altered: in ectopic beats P-wave is absent and in atrial fibrillation a continuous wave is observed instead of P-wave. Therefore, the progression of the cardiac cycle is adequately represented by the time relative to wave borders, that in turn become adequate reference points for estimation of medical information density in each individual heartbeat. Several algorithms were developed for automatic recognition and delineation of ECG waves and proven to perform with precision and accuracy acceptable for medical use. They can roughly be classified as signal derivative-, geometric template- or semantic sequence-based. The methods by Almeida et al. [21], by Martinez et al. [22], and by Dumont et al. [23] are based on the discrete wavelet transform (DWT). Other delineation methods reported use continuous wavelet transform (CWT) [24].

In this paper we propose a method for adaptive sampling of the electrocardiogram. The local sampling interval is driven by the expected conspicuity of the trace (i.e., medical relevance) calculated for a given progression of the cardiac cycle relative to its beginning. Therefore, a procedure for automated wave borders detection is necessary to control the piecewise projection of the gMRF to each particular heartbeat. The rest of this paper is organized as follows. In Section 2 the idea and processing scheme are presented together with a method for transforming the ECG trace conspicuity to an estimate of local information density, details on piecewise adaptation of the local relevance function, and ECG signal resampling. In Section 3 the evaluation of the method is reported with details on test signal set, error metrics and experiment results. In Section 4 a discussion is presented together with concluding remarks.

## 2. Materials and Methods

### 2.1. The Idea and Processing Scheme 

The key idea of proposed adaptive ECG sampling consists of allocating space in the output data stream accordingly to the information density in the input series (i.e., uniformly sampled ECG). Unlike in many signal compression methods or compressed sensing based on statistical description of information density, in our method the local information distribution is estimated on the background of perceptual studies [19,20]. Although an inverse transform does not bit-accurately reconstruct the regularly sampled ECG, the adaptive sampling preserves the most important sections of the heartbeat and maintains all diagnostic features of the original signal. Moreover, the method accepts various (e.g., application-specific) information distribution models arbitrarily specifying parts of the heart cycle as more or less important. Both scenarios have been experimentally proven and results are described in the later part of this paper. 

**Figure 1:**
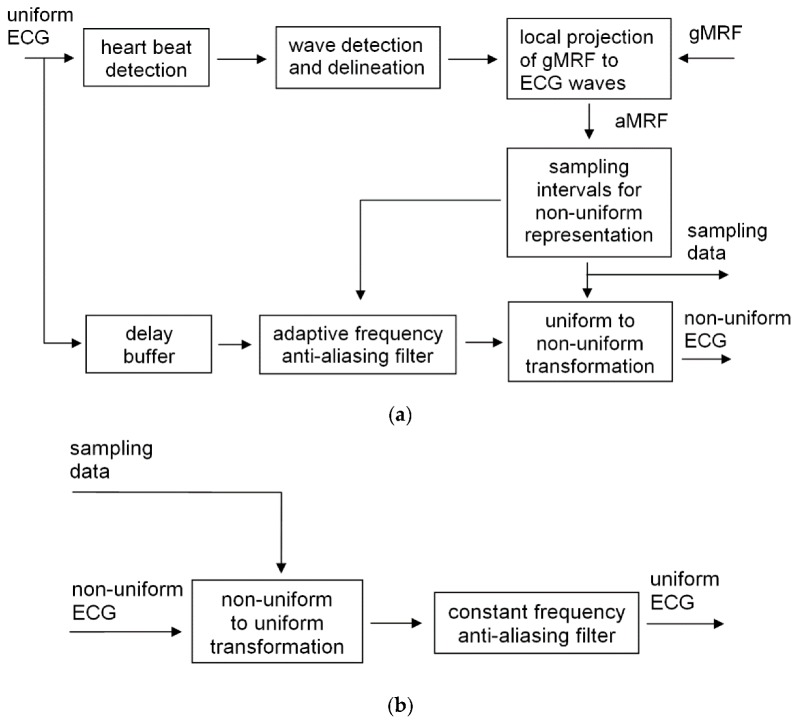
Block diagrams of the proposed adaptive sampling method (**a**) encoder, (**b**) decoder.

The adaptive sampling scheme (Figure 1) uses two procedures typical for the ECG interpretation-oriented processing chain: detection of heart beats (QRS complex) [25] and detection of ECG wave borders [21,22]. Both algorithms comply with the performance requirements for interpretive electrocardiographs [26], which guarantees high precision of wave borders delineation. Since both algorithms are designed to work with regular ECG signals, the proposed adaptive coding assumes uniformly sampled discrete time series at the input. Direct non-uniform sampling of analog signals (such as [17] or [27]) falls beyond the scope of this paper. 

The second step of the coding scheme is the local projection of gMRF to the ECG waves i.e., adaptation to the actual duration of consecutive electrocardiogram sections. The heart cycle rhythmicity is influenced by many factors of physiological origin: escape and premature discharges, conduction abnormalities, P-wave absence or multitude, simultaneous multi-center discharges, etc. [28]. Consequently, even if the wave order within a beat is predictable, the length of individual sections may vary independently in a broad range. As the description of expected data density is based on wave-related progression of the heart cycle, the gMRF has to be projected individually on each heartbeat. This operation described in detail in Section 2.4 yields the adapted medical relevance function (aMRF). 

The third step of the adaptive sampling scheme is the individual aMRF-based calculation of consecutive sampling intervals in the target irregular ECG. We used linear relationship (see Equation (4)), however other functions can also be employed. The sequence of sampling intervals determines the time points in which the ECG values are actually sampled (i.e., interpolated from the original discrete signal). Usually the number of intervals in the irregularly sampled representation is significantly lower than the number of original samples in the equivalent recording time. The sampling interval can be expressed by any real number, but for practical reasons we had to quantize the interval value. A high quantization step yields a concise representation of sampling rate at the price of spectral discontinuities causing signal distortions. A low quantization step guarantees good signal reproduction, but the sampling description consumes extra space. In the case of a multilead ECG record, the wave borders are determined jointly for all leads and stored in a single interval sequence. 

### 2.2. The ECG Trace Conspicuity as an Estimate of Local Information Density 

**Figure 2:**
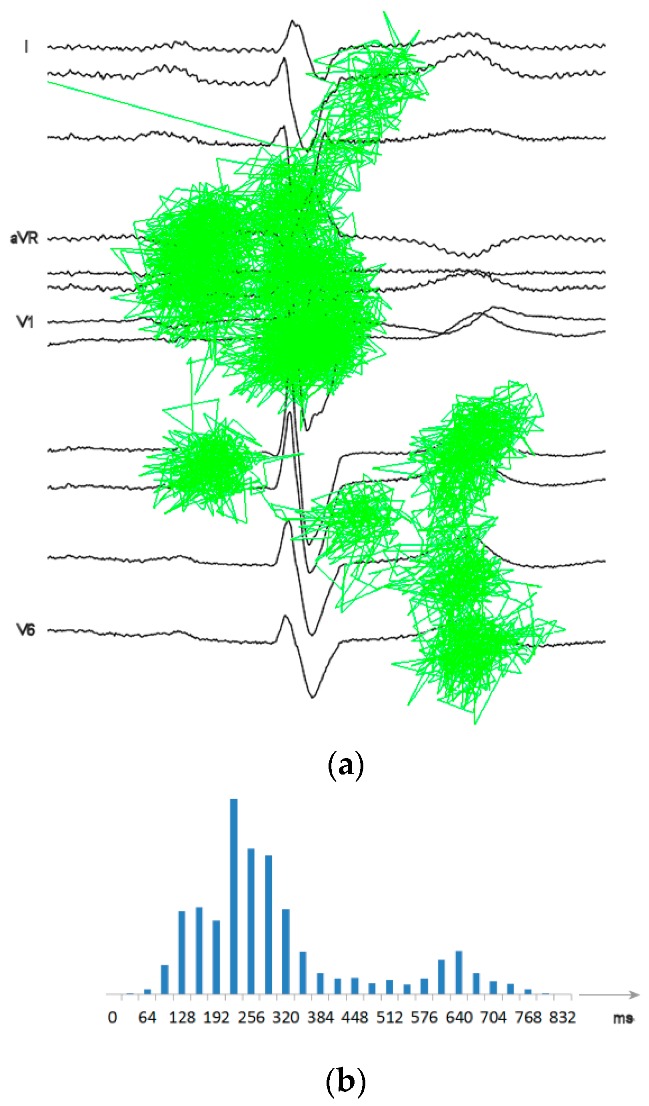
(**a**) The example of expert’s eyeglobe trajectory over a 12-lead ECG plot (CSE-Mo-001); (**b**) Corresponding bar graph of the attention density (each bar collects gaze time in a 32 ms interval in ECG record)—the background for gMRF (modified from [20]).

The proposed adaptive sampling scheme uses the gMRF as a quantitative estimate of local wave-related density of medical information. As it was proven with different scenes, visual interpretation of their content by a human observer involves alternating perception-cognition processes. Based on the relationship between the gaze time and the information amount, visual tasks are widely applied for objective assessment of preferences in graphic interfaces, advertising and many others [29,30]. Following these examples we studied ECG trace conspicuity and perceptual strategies of human experts for visual interpretation of records on display. The research presented in detail in [19] was focused on following the human expert reasoning and revealed different strategies of visual ECG interpretation for people with different degrees of expertise. Recorded eyeball trajectories were analyzed in the context of medical features represented in the displayed ECG traces (i.e., the wave borders were known but not displayed, Figure 2a). 

First, the ECG traces were section-wise normalized so as to equalize the length of corresponding waves from different records. Respective scanpaths were contracted or dilated accordingly. Next, gaze points were detected in the scanpaths and their center point horizontal coordinates were discretized with a step of 0.78 mm on display, corresponding to 32 ms of the ECG trace (at 25 mm/s). Finally, the time of gazing was collected in each ECG time slot separately and normalized to reveal interesting information about diagnostic data distribution (Figure 2b). As the histogram of the visual attention density was collected with respect to wave-dependent progression of the heart cycle, reference wave borders played a role of landmarks in the resulting gMRF.

The scanpath-based gMRF benefits from oculomotoric habits gathered by experts in their everyday practice, unconscious mutual perception-recognition interactions not affected by human intention, memory or verbalization limits. For these reasons the perceptual quantitative description of medical data distribution provides more objective assessment than any other method willingly controlled by the human. The perceptual model of the ECG proposed here as gMRF is universal since it was built from scanpaths of experts interpreting a wide range of normal and pathological records. Nevertheless, disease-specific models (i.e., specific medical relevance functions, sMRF) may also be built and applied accordingly to the status of particular patient. 

### 2.3. ECG Waves Delineation

The positions of wave borders are a background for individual calculations of the Medical Relevance Function for each heartbeat. As particular segments of a heartbeat independently vary in time, a piecewise projection of the gMRF does the necessary local stretching or contracting of the gMRF in order to fit its landmarks to the actual duration of consecutive cardiac events. 

Moreover, calculation of diagnostic parameters and comparing them between the original ECG and its counterpart reconstructed from the adaptive sampling, as well as the use of weighted diagnostic distortion (WDD) [5] (see Section 3.2) as a measure of signal reconstruction error, requires calculation of 18 diagnostic features by an ECG interpretive software. 

To this point we used a development version of Ascard 6 (^®^ by Aspel S.A., Zabierzow, Poland) software that meets the performance requirements of international IEC standard [26] and on the other hand allows for access to interpretation of metadata. The wave delimitation procedure embedded in Ascard 6 software uses the first and second derivatives of various versions of filtered signal in order to determine the point where the wave energy emerges from the background noise [31]. The algorithm also uses wave-specific features individual for each wave’s onset and endpoint.

The Common Standard for Quantitative Electrocardiography (CSE) database, used in the experimental part for local adaptation of gMRF, provides reference wave border points of a representative heartbeat of each record [32] (see Section 3.1). The opportunity was taken to check whether substituting the values calculated with Ascard 6 software by their reference counterparts influences the adaptive ECG sampling result.

### 2.4. Piecewise Adaptation of the Local Relevance Function

Assigning *k* = {1 … 5} to the consecutive borders of waves: P-onset, P-end, QRS-onset, QRS-end and T-end (T-onset is not considered as standard fiducial point), the projection of gMRF to aMRF consists in calculating the values of the latter,

(1) $$\underset{b\in aMRF}{\forall }\;\underset{a\in gMRF}{\exists }:aMRF(b)=gMRF(a)$$

where *a* and *b* express integer sample numbers in adapted and generalized medical relevance functions, respectively, and

(2) $$a=\frac{\left(a_{k}-a_{k-1}\right)\times \left(b-b_{k-1}\right)}{\left(b_{k}-b_{k-1}\right)}+c$$

where *c* is a complement transferring fraction of border sampling interval between ECG waves, i.e.,

(3) $$c=\{\begin{array}{c} \frac{a_{k+1}-a_{k}}{b_{k+1}-b_{k}}\\ 0\\ \frac{a_{k-2}-a_{k-1}}{b_{k-2}-b_{k-1}} \end{array}\begin{array}{c} for\;k=2\\ for\;k\in \left\{3,4\right\}\\ for\;k=5 \end{array}$$

**Figure 3:**
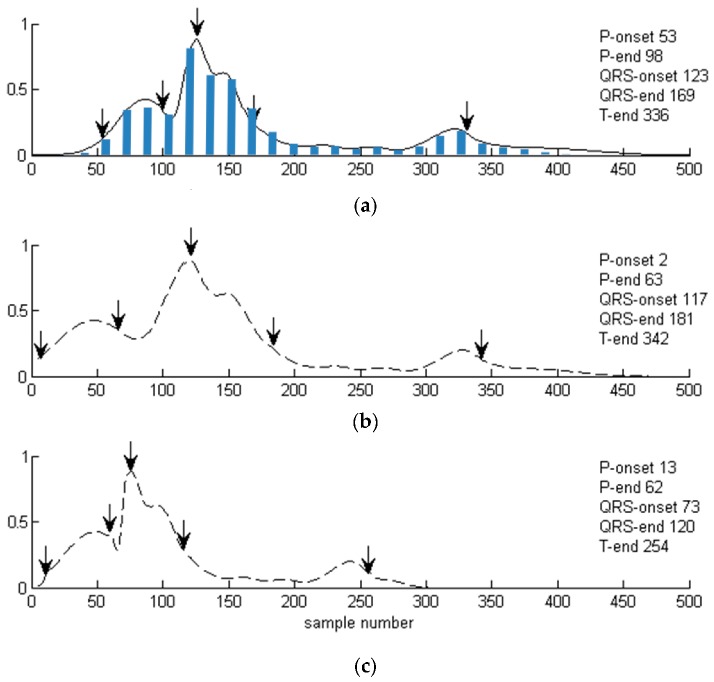
Projection of generalized medical relevance function (gMRF) to local positions of the heartbeat sections: (**a**) gMRF (with the bar graph it stems from), (**b**) aMRF (adapted medical relevance function) calculated for CSE-Mo001 record, (**c**) aMRF calculated for CSE-Mo003 record.

We applied the piecewise linear projection of gMRF to aMRF (Figure 3) for its computational simplicity and without noticing any consequences of singularities in aMRF caused by stepwise changes of gMRF sampling. Otherwise, either digital filtering of the resulting aMRF or projection with the use of cubic splines with nodes at the landmarks are possible alternatives. All calculations use real number representation in time and value domains of these functions. 

Finally, for each heartbeat the values of the aMRF are used to control the local sampling interval *ls*(*t*) within the range corresponding to frequency limits (*f_m_*, *f_s_*) accordingly to the linear relationship:

(4a) $$ls\left(t\right)=T_{m}+\left(T_{s}-T_{m}\right)\times aMRF\left(t\right)$$

or conversely,

(4b) $$ls\left(t\right)=\frac{1}{f_{m}}+\frac{f_{m}-f_{s}}{f_{m}\times f_{s}}\cdot aMRF\left(t\right)$$

**Figure 4:**
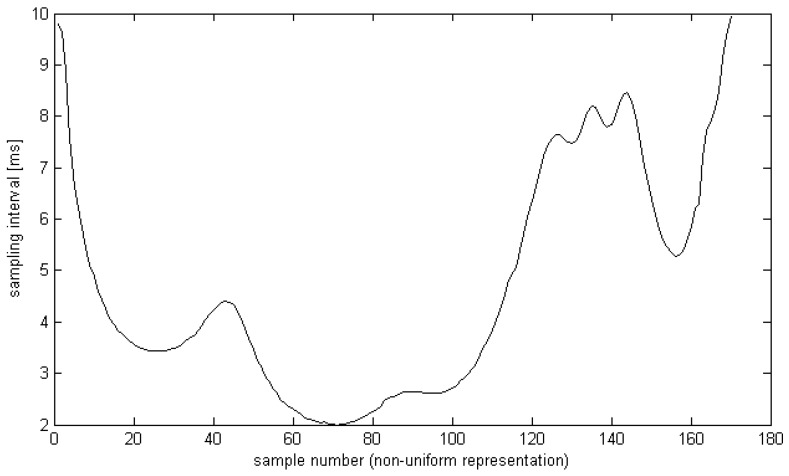
Interval of consecutive samples in a non-uniform representation (*f_m_*_1_ = 100 Hz) calculated for the reference beat from file CSE001.

In the proposed implementation, the adaptive algorithm is dedicated to the ECG signal sampled at *f_s_* = 500 Hz and the minimum usable value of local sampling frequency tested in two experiments was set to *f_m_*_1_ = 100 Hz (Figure 4) and *f_m_*_2_ = 50 Hz respectively. 

### 2.5. ECG Signal Resampling 

The objective of the sampling problem is to recover a function *f* on $R^{d}$ from its samples {*f*(*x_j_*): *j* ∈ *J.*}, where *J.* is a countable indexing set, and *f* satisfies some a priori constraints [33]. Extension of the classical Shannon theory to the non-uniform sampling of bandlimited functions specifies that for the exact and stable reconstruction of such function *f* from its samples {*f*(*x_j_*): *x_j_* ∈ *X*}, it is sufficient that the Beurling density,

(5) $$D(X)=\underset{r\to \infty }{\lim}\;\underset{y\in R}{\inf}\frac{\#X\cap \left(y+\left[0,r\right]\right)}{r}$$

(where *r* is radius of sampling grid and *y*—sample position), satisfies *D*(*X*) > 1 [34,35]. Conversely, if *f* is uniquely and stably determined by its samples on *X* ⊂ R, then *D*(*X*) ≥ 1. 

Solution of the sampling problem *f* in non-uniform shift-invariant bases $V_{v}^{p}(\phi )$ (where *p* is space dimension, *ν* is the weight function and *ϕ* is the space generator) consists of two parts.

- Given a generator *φ*, conditions on *X* have to be defined, usually in the form of a density, such that the norm equivalence (6) holds.
  (6) cp‖f‖Lvp≤(∑xj∈X|f(xj)|p|v(xj)|p)1p≤Cp‖f‖Lvp
  Then, at least in principle, $f\in V_{v}^{p}(\phi )$ is uniquely and stably determined by ${f|}_{X}$.
- Reconstruction procedures useful and efficient in practical applications have to be designed as fast numerical algorithms which recover *f* from its samples ${f|}_{X}$, when (6) is satisfied.

Since the iterative frame algorithm is often slow to converge and its convergence is not even guaranteed beyond *V*^2^(*φ*), alternative reconstruction procedures based on Neumann series have been designed [34]. In [33] Aldroubi presented the example iterative algorithm with the proof of the convergence of results. The reconstruction of the uniform biosignal from an incomplete time series was also developed by Candes et al. [36] and Needell and Tropp [37]. 

In the proposed algorithm for adaptive ECG sampling we used the cubic splines interpolation to transform the ECG from its native uniform representation to the adaptively sampled representation and vice-versa. Considering the uniform representation as a particular case of non-uniform time series, the approximation first projects the input time series *N_j_*({*n*, *v*(*n*)}) to the continuous space with the use of 3rd order polynomial function,

(7) $$S_{n}\left(t\right)=a_{n}+b_{n}(t-t_{n})+c_{n}{\left(t-t_{n}\right)}^{2}+d_{n}{\left(t-t_{n}\right)}^{3}$$

where *t* ∈ [*t_n_*, *t_n_*_+1_], *n* ∈ {0, 1, … *N* − 1} is best fitted to the time series *N_j_*. Next, the output signal representation is obtained by sampling the *S_n_*(*t*) at desired time points *m*:

(8) $$N_{j}^{\prime }\left(m\right)=\underset{m}{\sum }S_{n}\left(t\right)\times \delta \left(t-m\times ls\left(t\right)\right)$$

**Figure 5:**
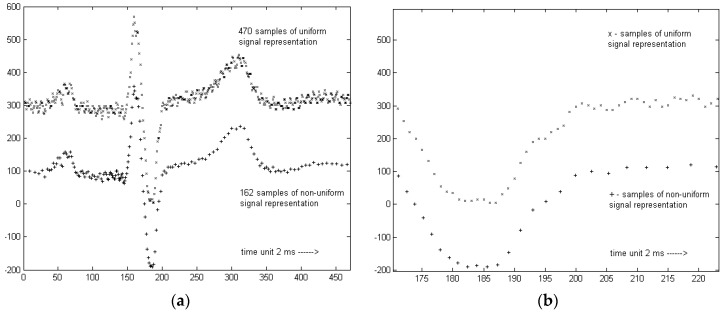
Regular and irregular representations of the same heartbeat (**a**) global scale, (**b**) local scale (terminal section of QRS). Vertical axes represent the ECG voltage, approximately 2.44 μV per unit.

In the case of forward transformation, the positions of input sampling points *n* are equispaced whereas the positions of output sampling points *m* are determined by the local sampling interval *ls*(*t*) (see Equation (4), Figure 5). In the case of inverse transformation, the input time series comes as non-uniformly sampled, and considering the information about local distances between samples, the cubic splines interpolation yields the uniform ECG representation.

### 2.6. Implementation Details

For the purpose of experimental assessment of our method, the adaptive ECG sampling algorithm was implemented in C++ on a Windows-based PC platform, however, due to moderate complexity, it may be easily transferred to a portable or wearable device with floating-point arithmetic. Heartbeat detection and wave delineation procedures were used without modification from the firmware of Ascard 6 bedside interpretive electrocardiograph. The accuracy of wave delineation is not critical due to a smooth shape of aMRF. Apart from the cardiology-oriented procedures, second contribution to the computational complexity comes from the translation of uniform to non-uniform representation. The use of cubic splines-based interpolation instead Aldroubi iterative algorithm reduces the calculation costs.

The finite impulse response (FIR) filter with 6 dB/octave (i.e., 20 dB/decade) slope has been used for low-pass anti-alias filtering of discrete uniform ECG. Unlike a regular design where the number of taps weighting the sequence of delayed input samples is fixed, we used a tunable fractional-delay filter. Respective theory and literature review are presented in [38] or [39], and such filters are also available in a recent Matlab toolbox and available as FPGA implementations [40]. As the result of exploring different design variants, we selected the filter order *N* = 10 which was the best compromise between cut-off frequency tuning range, phase flateness degree, and computational cost. Anti-alias filtering is directly followed by signal resampling. This procedure uses regular cubic splines interpolation, which is known as optimally matching two differently sampled signals and avoids discontinuities in the sampling rate function. 

The sampling interval information (see Equation (4a), Figure 4) was collectively used for all simultaneous leads. Its value was quantized to 6 bits, yielding *P* = 64 possible values linearly assigned to durations of sampling interval so as $ls=T_{s}+{\frac{p}{64}}_{}(T_{m}-T_{s})$ i.e., between 2 ms and 10 ms with a 0.125 ms step (or between 2 ms and 20 ms with a 0.281 ms step).

## 3. Evaluation of the Method 

The proposed method has been implemented and validated in an experimental environment Matlab (MathWorks, Natick, MA, USA) with two external executable procedures compiled from C++ source. The evaluation procedure follows a common scheme and includes:

1. Selecting a set of test signals complying with international standards (the CSE Database);
2. Selecting tools (the ECG interpretive software) and error measures (PRD, local PRD and WDD);
3. Selecting the range of method parameters (gMRF, sMRF, sampling interval);
4. Modifying signals from the test set with the proposed adaptive sampling method in all combinations of parameters by encoding the original (see Figure 1a) and decoding the encoded (see Figure 1b) records;
5. Comparing differences between original and processed records with error measures (PRD compares discrete values of samples, whereas the WDD compares values of diagnostic results);
6. Statistical processing of error values estimated for each file with each combination of parameters.

Steps 1–3 are described in detail in Section 3.1, Section 3.2 and Section 3.3.

### 3.1. The Test Signal Set 

The proposed adaptive ECG sampling algorithm was tested with the use of CSE Multilead Database [32] recommended for electrocardiographs performance tests by the IEC [26]. It is worth a remark that the CSE Database is used for industrial validation of ECG wave delineation accuracy, and the perceptual model is adapted to the information on wave start and endpoint positions. Consequently, tests with the CSE files are sufficient for a complete evaluation of the method and the algorithm performance in the case of ectopic or missing beats, arrhythmias, etc., can be inferred from the reliability of wave border detection in those cases. 

We used 125 automatically annotated 12-lead signals from CSE Dataset 3. The Dataset 3 consists of proportionally represented examples of normal ECGs, myocardial infarction, bundle branch blocks, premature ventricular contractions, ischemic ST changes, atrial fibrillation and many others. It is sampled with a 12-bit resolution at 500 samples per second to a data stream of 72,000 bits per second (bps). The average additional data stream carrying the local sampling rate is 1034 bps (i.e., 1.44% of the original record’s data volume). 

We first compared adaptive ECG sampling results for representative heartbeats of each CSE record with use of either their reference or the Ascard 6-calculated wave border values. Although the Ascard 6 software (see Section 2.3) is certified, the respective fiducial points differ by up to 2–4 ms depending on the point type and the presence of noise. Substituting these values was possible with just writing a few lines of I/O instructions to the development version of the Ascard 6 software. With any of the 123 CSE files (pacemaker-originated records 67 and 70 were discarded for the lack of annotation) we did not notice any difference of sampling results. Consequently, we stated that the inaccuracy of wave delineation procedure within Ascard 6 software is not significant and can be neglected. In all remaining tests, additionally taking into account beat-to-beat variability in each 12 s ECG sequence, individual wave borderlines were determined with the Ascard 6 software.

### 3.2. The Error Metrics 

In order to make our results comparable with existing methods, we use the PRD (Equation (9)) and data compression ratio CR as a first-step approach to the assessment of transparency of proposed adaptive ECG sampling:

(9) $$PRD=\sqrt{\frac{\sum_{i=1}^{n}{\left[x_{1}\left(i\right)-x_{2}\left(i\right)\right]}^{2}}{\sum_{i=1}^{n}{\left[x_{1}\left(i\right)\right]}^{2}}}\cdot 100\%$$

However, due to intrinsic inadequacy of PRD that averages all errors in time without consideration of their position with regard to medical information, we also calculated its values separately for each ECG wave, as suggested in [1]. This method is reported as a local distortion measure that depends on estimated ECG sections and possible weighting of their significance [41]. The distortion level was also provided in microvolts as an average peak-to-peak error value, for the reason of compatibility with performance requirements for interpretive electrocardiographs [26]. 

To avoid the abovementioned flaw of the PRD, we applied a more comprehensive error metric based on diagnostic features to reveal differences of primary diagnostic outcomes derived from the original and adaptively sampled ECG record. A widely discussed, but yet recently applied global quality estimate based on comparing the PQRST complex features of the two ECG signals is the weighted diagnostic distortion (WDD) introduced in [5]. It was defined as

(10) $$WDD\left(\beta ,\hat{\beta }\right)=\Delta \beta^{T}\frac{\Lambda }{tr\left(\Lambda \right)}\Delta \beta \cdot 100$$

where Δ*β* is the normalized difference vector between original and processed PQRST features, where *β* and $\hat{\beta }$ represent two vectors of 18 diagnostic features (RR_int._, QRS_dur._, QT_int._, QTp_int._, P_dur._, PR_int._, QRS_peaks_no._, Q_wave_exsist._, Δ_wave_exsist._, T_shape_, P_shape_, ST_shape_, QRS(+)_amp._, QRS(−)_amp._, P_amp._, T_amp._, ST_elevation_, ST_slope_) of compared beats and Λ is a diagonal matrix of weights heuristically set to [42],

Λ = diag [2.5 2.5 1 1 2 2 1 0.5 0.1 1.5 1 3 1.5 1.5 1 1 3 3](11)

This ECG-specific error metric, although requiring interpretive calculation of diagnostic outcomes, yields results related to medical findings equivalence rather than to signal representation accuracy. Therefore it is more adequate to evaluate the medical content preservation in signals with irregular distribution of information. Since the definition of WDD roughly reflects the local density of medical data in the ECG, expressed by diagnostic parameters, we consider it as the principal quality estimator of proposed adaptive ECG sampling. Moreover, the use of WDD shifts the evaluation of our adaptive sampling method from the signal domain to the parameter domain and thus reliably reflects the possible alteration of medical content. A serious cost of this is the necessity of using an interpretive software, of which none shows a 100% accuracy. 

### 3.3. Performance Assessment 

**Figure 6:**
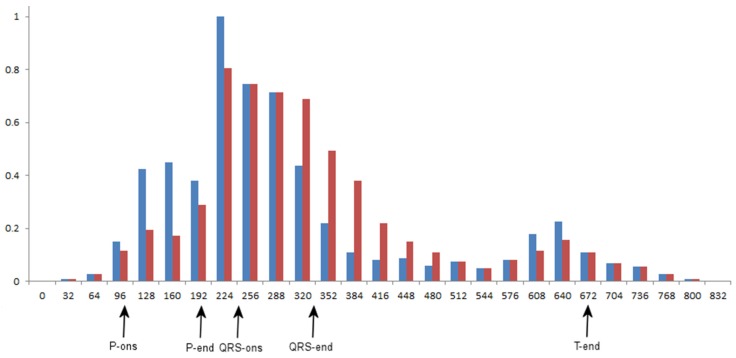
Comparison of bar graphs for scanpath-determined gMRF (blue) and example infarct or a conduction defect-oriented sMRF (red). Wave borders are: P-onset: 106, P-end: 196, QRS-onset 246, QRS-end: 338 and T-end 672.

With the aim of exploring the possible flexibility of the adaptive ECG sampling, we applied two different ranges of sampling frequency adaptation: (1) 100 Hz … 500 Hz and (2) 50 Hz … 500 Hz. We also used two different medical relevance functions. Besides the original gMRF, obtained directly from scanpath studies (see Figure 2b), we simulated a specific medical relevance function (sMRF) with a purpose-oriented region of interest (ROI) focused on the end of QRS complex (e.g., for the investigation of an infarct or a conduction defect [29]). This variant of sMRF emphasizes the relevance and increases the accuracy of QT section at the price of saving samples mostly in P-wave vicinity (Figure 6). 

Results of experiments for compression, distortions and medical parameter differences are presented in Table 1.

## 4. Discussion

The presented adaptive ECG sampling algorithm, although not bit-accurate, shows interesting compression efficiency, making it worth considering in clinical applications. It may be classified as an alternative to bit-accurate methods yielding the compression ratio in the order of 3 at the price of high computational complexity [10] to the quality-on-demand (lossy-to-lossless) algorithms [3,43] and to recently proposed compressed sensing [13,15] or adaptive sensing [16,17] methods. The main advantage of the newly proposed algorithm is that unlike methods in the last two categories, the temporal distribution of distortions is based on medical rather than on statistical features of the signal (Table 1). For this reason, the quality estimate based on primary ECG diagnostic parameters (WDD) shows only little difference caused by the transformation.

We do not follow the example of several authors [3,7,42,44,45,46,47,48] using the MIT-BIH database [49] for tests of adaptive ECG sampling technique. The reason is threefold:

The sampling frequency of the MIT-BIH database is too low; following the paradigm typical for long-term recording, sampling at 360 Hz avoids oversampling in low-frequency components, limits the bandwidth of the high-frequency sections, and reduces the interval range (*f_s_–f_m_*) available for adaptive sampling.

The CSE Database provides reference positions of wave border points that enable evaluation of whether the proposed algorithm is robust to possible inaccuracy of wave delimitation.

The proposed algorithm uses the aMRF commonly calculated and stored for all leads in a multidimensional signal, therefore its efficiency decreases with the leads number.

A broad discussion was held with collaborators and experts in the field whether to apply the proposed method to the MIT-BIH standards, namely arrhythmia and compression databases. This would allow us to compare the proposed method to the values reported by Sayadi et al. [42] on that dataset or to compare the compression efficiency to the variety of algorithms like the ones proposed by Fira et al. [45] or Kim et al. [50] who reported the quality score (QS) above 15 for the MIT-BIH database. For three reasons we decided not to follow this thinking.

The proposed method is not yet another data compression algorithm—its novelty consists in the use of a priori knowledge about the ECG content derived from the human perception instead of local statistics of the signal. 

The use of reference wave border points provided by the CSE database makes it possible to prove the robustness of the coding method to the inexact performance of the delineation software.

The QS proposed as a ratio of CR to PRD [45] inherits the principal drawback of the PRD that is the negligence of local variations of ECG signal relevance.

Additionally, as we are skeptical of the PRD as a medically-justified measure of distortion and propose using the WDD instead, we need a diagnostic software in order to calculate all 18 heartbeat features given in Equations (10) and (11). Following [51] we also did preliminary tests with the European QT Database providing reference wave borders available from Physionet at no charge. The database consists of 2-lead ECG signals, and we tried to simulate the missing leads in order to feed them to Ascard 6 12-lead diagnostic software. At this point we had to give up since the results we got for wave delineation were significantly different from the values given in the database. 

Parkale and Nalbalwar give a complete survey of the CS techniques in [52]. The main performance results of the most significant methods are summarized in Table 2 for two scenarios: Scenario 1, distortion-optimized (with reasonable compression ratio), and Scenario 2, compression-optimized (with acceptable distortion level).

Comparing our work to the CS algorithms one should note different approaches to testing the quality of the output ECG. Mamaghanian et al. [14] and other followers refer to the paper by Zigel et al. [5] in specifying the ‘quality class’ as ‘very good’ for PRD < 2% or ‘good’ for 2% < PRD < 9%. For Luo et al. [55] the ECGs recovered with PRD < 6% are ‘essentially undistorted’ and Craven et al. [13] allow for PRD > 5% for ‘clinically relevant metrics’ but, despite the title, they focus on heart rate variability (HRV) parameters, more tolerant to amplitude distortion.

In works by Rieger et al. [16], Kim et al. [56], and Yazicioglu et al. [17], the bi-frequency sampling is controlled by the algorithm recognizing high activity and low activity sections. In [16] the ECG trace has to meet given peak and curvature conditions (i.e., first and second derivatives are calculated) to switch basic sampling frequency 50 Hz to the fast rate of 400 Hz. The threshold was set to effectively recognize the QRS beginning, maximum and end points in normal heartbeats. As a result, the data compression ratio of 1.6 was achieved. In [56], following the wavelet-based detection of QRS the basic sampling frequency of 64 Hz is stepwise raised to 512 Hz. As a result the data compression ratio of 4.5 was achieved. In [17] the activity detector circuit senses the rate of change of the input signal by using a switching capacitance differentiator. As the activity passes a given threshold, the basic sampling frequency of 64 Hz is stepwise raised to 1024 Hz. The data compression ratio depends on the duty cycle controlled by the value of activity threshold. For duty cycles in the range of 4–12%, the compression ratios of 9.94–5.72 are expected respectively. In all these works authors focus on the circuitry design and power efficiency rather than on ECG signal diagnostability, and thus do not provide information on the ECG distortion level. Despite a quantitative comparison of these methods with our work not being possible, they share a common concept of non-uniform distribution of medical information in the ECG. Therefore, the gMRF proposed in this paper can be seen as an advanced version of ‘activity detector’ controlling the actual ECG sampling in 64 steps instead of 2, which allows us to preserve best signal quality within the ECG waves. 

A bi-frequency compression scheme based on local sub-sampling (decimation) of the signal is also recommended by the SCP-ECG communication protocol (ENV 1064) [57]. In our approach, the sampling interval is adjusted in a nearly continuous way without precisely distinguishing bordered zones. This provides a fair tolerance margin for the accuracy of waves delineation. 

Aiming at future industrial implementation, we thoroughly checked the temporal distribution of distortions and their possible influence to the diagnostic result. The global PRD value (Table 1) seems to be high, but it is noteworthy that within the waves the distortion level in terms of wideband noise meets the requirements of industrial IEC standards [26] (25 μV, accordingly to 51.106.4) and is very close to quantization error requirements (5 μV, accordingly to 51.107.4). The duration of intervals is also little affected by the adaptive sampling, and extending the sampling frequency to as low as 50 Hz together with application of a QRS-focused region of interest still yields results acceptable by the IEC standard (marked by a double star in Table 1). 

CS methods are often presented in the context of computational efficiency, particularly stressed in low-power wireless networks of sensing nodes. Although our primary goal is the quality of medical content, we estimated the computational complexity of the method by implementing the detection and wave delineation parts of the Ascard 6 software to a mobile platform with PXA-270 CPU running at 624 MHz with 64 MB of operation random access memory and 32 MB of flash memory under Linux OS. Additionally, tunable low-pass filters and cubic splines-based procedures for translation of uniform to non-uniform ECG representation were also implemented in the platform. All the evaluation procedures (e.g., WDD) were kept on the PC and used offline. Since the adaptive sampling is conceived as a continuously running procedure, the results on processing time are expressed as a percentage of real ECG duration as follows:

- On a PC platform i7 3770 (^®^ Intel, Santa Clara, CA, USA), 3400 MHz, 8 GB RAM—0.943% (i.e., 106 times faster than the ECG acquisition);
- on a mobile platform PXA-270 (^®^ Toradex AG., Horw, Switzerland), 624 MHz, 64 MB RAM—7.518% (i.e., 13.3 times faster than the ECG acquisition).

The gMRF is derived as a result of the pursuit for local conspicuity of the ECG trace and represents common perceptual habits of cardiology experts participating in our visual experiment [19]. This function generalizes the knowledge from cardiology expert perception of the ECG trace and reflects the local relevance of the signal that would be difficult to express in another way. This relationship can easily be modified in order to create various application-oriented or user-tailored profiles, differing by temporal allocation of regions of interest and the remaining zones where distortions are tolerable. An example of such approach was also tested (sMRF) and yielded a promising compromise of coding efficiency to distortion ratio (CR = 4.72, PRD = 4.88, WDD = 0.41).

The gMRF, being an experimental perception-derived relevance curve, has been applied to modulating the ECG sampling. For this task alternative sMRFs may be developed and used accordingly to a specific medical purpose. Since the shape of the sMRF is a principal factor imposing the compression efficiency, a question arose as to whether one could predict the CR from the sum of MRF bins (ΣMRF). In the particular case of CSE Database records, accompanied by reference specifications of the length of each particular heartbeat, thanks to the performed temporal normalization such a strict relationship can be proven. In this case the output data stream *d* may be approximated as

(12) $$d=\left[f_{m}+\left(f_{s}-f_{m}\right)\times bl\times \underset{b}{\sum }MRF\left(b\right)\right]\times sr$$

where *bl* stands for bin length [s] (in our work being equal to 0.032 s), *b* is the bin number, and *sr* is the sample resolution (equal to 12 bits per sample).

In a general case, such a precise estimate cannot be calculated due to the presence of additional factors. Specifically, the CR will be higher than estimated by ΣMRF in case of slow rhythm (long T-P interval) due to prolonged use of *f_s_* between the adjacent heart beats. Otherwise, the CR will be lower than estimated by ΣMRF in the case of fast rhythms because main shortenings in the ECG pattern take place out of the waves (i.e., the accelerating heart first reduces its inactivity periods). A separate approach should be studied in cases of abnormal ECG when wave borders cannot be determined. 

The proposed adaptive sampling technique makes sole use of local signal oversampling and therefore does not pretend to compete with existing ECG compression methods. In irregularly sampled ECG series, a significant short time (i.e., sample-to-sample), long time (i.e., heartbeat-to-heartbeat) and spatial (i.e., lead-to-lead) correlation is preserved. Therefore, further improvement of sampling efficiency is expected as a result of combining the adaptive sampling with long-term prediction technique (the use of beat-to-beat similarity of the ECG) and/or with ECG leads decorrelation (reduction of signal dimensionality) considered in future versions. In our opinion, this kind of sampling may have a similar impact on the telemedicine of tomorrow as the perceptual coding had on the audio and video broadcasting techniques of today.

## Figures and Tables

**Table 1:** Data compression, distortions and medical parameter differences for adaptive ECG sampling.

Parameter		gMRF (Perceptual)		sMRF (with ROI)	
*f_s_* Range (Hz)		100 … 500	50 … 500	100 … 500	50 … 500
Compression ratio		3.01	3.61	3.83	4.72
PRD [%] ([μV] *)	global	3.11 (46.6)	3.73 (55.9)	3.96 (59.3)	4.88 (73.1)
	within P-wave	0.16 (2.4)	0.18 (2.7)	0.35 (5.3)	0.41 (6.2)
	within QRS complex	0.22 (3.3)	0.22 (3.3)	0.22 (3.3)	0.24 (3.6)
	within T-wave	0.37 (5.6)	0.41 (6.2)	0.44 (6.7)	0.47 (7.1)
	out of waves	1.11 (16.6)	1.77 (26.5)	2.70 (40.4)	3.93 (58.8)
WDD [%]		0.21	0.23	0.37	0.41
RR interval std [ms]		1.5	1.5	1.5	1.5
P-wave duration std [ms] (15) **		10.3	10.7	12.4	14.1
PQ interval length std [ms] (10)		7.1	7.2	8.6	9.7
QRS duration std [ms] (10)		7.6	7.6	7.6	7.6
QT interval length std [ms] (30)		14.7	16.5	16,1	18.2
P axis std [deg]		7.5	8.8	7.8	9.7
QRS axis std [deg]		2.1	2.7	2.4	3.0
T axis std [deg]		3.1	3.5	3.3	3.5

* acceptable value specified in IEC 60601-2-51 is 25 μV or 5% for amplitudes above 500 μV. ** acceptable value of standard deviation with reference to CSE Database results specified in IEC 60601-2-51.

**Table 2:** Performance of the proposed adaptive sampling method compared to recent landmark systems for ECG compressed sensing; NB authors use different data sets for testing.

Work (Test Set)	Method	Scenario 1		Scenario 2	
		CR	PRD [%]	CR	PRD [%]
Mamaghanian [14] (MIT-BIH)	wavelet db10	3.70	2.00	10.00	9.00
	CS	2.04	2.00	3.45	9.00
Mishra [53] (10 ECGs custom set)	db	2.00	1.31	6.00	17.37
	rbio3.9	2.00	0.32	6.00	10.84
Craven [13] (MIT-BIH)	SPIHT	6.10	1.95	12.00	4.00
	AD-Q6	6.75	3.20	11.10	4.50
Polania [15] (subset of MIT–BIH)	MMB–IHT	6.40	3.76		
	MMB–CoSaMP	6.40	3.96		
Polania [51] (European QT DB)	RBM-OMP-like	2.00	1.20	5.00	6.30
	BPON	2.00	1.30	5.00	8.90
Chen [54] (MIT-BIH)	rbio5.5	2.00	10.03	5.00	46.63
	rbio5.5-JBHI	2.00	3.85	5.00	9.10
this work (CSE)	**perceptual**	**3.01**	**3.11**	**4.72**	**4.88**
